# GWAMA: software for genome-wide association meta-analysis

**DOI:** 10.1186/1471-2105-11-288

**Published:** 2010-05-28

**Authors:** Reedik Mägi, Andrew P Morris

**Affiliations:** 1 Genetic and Genomic Epidemiology Unit, Wellcome Trust Centre for Human Genetics, University of Oxford, Oxford OX3 7BN, UK; 2 Oxford Centre for Diabetes, Endocrinology and Metabolism, University of Oxford, Churchill Hospital, Headington, Oxford OX3 7LJ, UK

## Abstract

**Background:**

Despite the recent success of genome-wide association studies in identifying novel loci contributing effects to complex human traits, such as type 2 diabetes and obesity, much of the genetic component of variation in these phenotypes remains unexplained. One way to improving power to detect further novel loci is through meta-analysis of studies from the same population, increasing the sample size over any individual study. Although statistical software analysis packages incorporate routines for meta-analysis, they are ill equipped to meet the challenges of the scale and complexity of data generated in genome-wide association studies.

**Results:**

We have developed flexible, open-source software for the meta-analysis of genome-wide association studies. The software incorporates a variety of error trapping facilities, and provides a range of meta-analysis summary statistics. The software is distributed with scripts that allow simple formatting of files containing the results of each association study and generate graphical summaries of genome-wide meta-analysis results.

**Conclusions:**

The GWAMA (Genome-Wide Association Meta-Analysis) software has been developed to perform meta-analysis of summary statistics generated from genome-wide association studies of dichotomous phenotypes or quantitative traits. Software with source files, documentation and example data files are freely available online at http://www.well.ox.ac.uk/GWAMA.

## Background

Genome-wide association (GWA) studies of hundreds of thousands of single nucleotide polymorphisms (SNPs), genotyped in samples of thousands of individuals, such as those undertaken by the Wellcome Trust Case Control Consortium [1], have proved successful in identifying novel common variants contributing moderate effects to a wide range of complex human traits (odds ratios greater than 1.2 for dichotomous traits or heritability of at least 1% for quantitative phenotypes). However, much of the genetic variation underpinning variation in these traits remains, as yet, unexplained. One natural way to increase power to detect rarer variants of more modest effect is to increase sample size. This can most readily be achieved through meta-analysis of multiple studies from the same or closely related populations, increasing the sample size to the order of tens of thousands. Such analyses have led to the identification of multiple, now established associations that would not otherwise have been identified in any individual study [2-4].

Meta-analysis of GWA studies has been greatly assisted by the development of imputation techniques [5,6] which predict genotypes not directly typed on available GWA genotyping products, but which are present on a dense reference panel of haplotypes, such as those available as part of the International HapMap Project [7] or the 1,000 Genomes Project [8]. With this approach, the results of GWA studies can be combined through meta-analysis of millions of SNPs, even if samples are interrogated with different GWA genotyping products.

The statistical methodology underlying meta-analysis is already well established [9], and freely available software packages provide routines for its implementation [10]. However, in the context of GWA studies, we face a number of unique challenges that make these existing tools impractical: (i) results are often combined for many studies for millions of SNPs, thus requiring memory efficient data manipulation; (ii) there may be over-dispersion of GWA test-statistics due to population structure, and between study variation, both of which must be accounted for in the meta-analysis; and (iii) computational difficulties in combining results obtained using different GWA genotyping products which may be aligned to different strands.

To address these challenges, we have developed the GWAMA (Genome-Wide Association Meta-Analysis) software to perform meta-analysis of summary statistics generated from GWA studies of dichotomous phenotypes or quantitative traits. The software incorporates tools to align studies to the same reference strand, irrespective of the GWA genotyping product, where possible, and optionally performs genomic control [11] of summary statistics to correct for population structure within each study, and potential variation between studies. The software also incorporates scripts for the generation of summaries of genome-wide meta-analyses including Manhattan and quantile-quantile (QQ) plots. Here, we demonstrate application of the GWAMA software to meta-analysis of 5 GWA studies, typed using different GWA genotyping products, but imputed at more than 2.3 million SNPs present on the International HapMap Project reference panels [7]. There are already several software packages available for meta-analysis and therefore comparison with some of the most widely used programs is part of current study.

## Implementation

Consider a meta-analysis of *N *GWA studies, not necessarily typed using the same genotyping product or imputed to the same reference panel. We assume that studies have been filtered for appropriate quality control metrics to exclude poorly genotyped or imputed SNPs [12]. For each study, the following information is required for each good quality SNP: (i) the marker identifier; (ii) the allelic effect estimate and corresponding standard error (or an allelic odds ratio and 95% confidence interval in the case of a dichotomous trait); and (iii) the allele for which the effect has been estimated and the complimentary non-reference allele. Optionally, users may provide: (i) the frequency of the reference allele and the strand to which it has been aligned, which may aid alignment of AT/GC SNPs; (ii) the sample size contributing to the effect estimate; and (iii) an indicator to identify if the SNP has been directly genotyped in the study or imputed from a reference panel.

GWAMA begins by aligning all studies to the same reference allele at each SNP. If strand information is provided, a log file records potential misalignments and any corrections made based on the provision of reference alleles. Fixed effects meta-analysis is then performed for each SNP by combining allelic effects weighted by the inverse of their variance. The software performs tests of heterogeneity of effects across studies, and reports simple summaries of the direction of their effect in each to highlight potential inconsistencies in results. In the presence of heterogeneity of effects between studies, GWAMA can perform random-effects meta-analysis for each SNP by calculating the random-effects variance component. Graphical summaries of the results of the meta-analysis can be generated using the output of GWAMA, in conjunction with accompanying R scripts [10], provided that a map file containing SNP identifiers, chromosome and location are specified. A dense map file is provided with the GWAMA software which includes SNPs incorporated on a wide range of GWA genotyping products and variants present on the Phase 2 HapMap reference panel [7].

### File formatting prior to meta-analysis

GWAMA is distributed with PERL scripts to format output from GWA association tools including PLINK [13] and SNPTEST [14]. The scripts extract the appropriate summary statistics from the output of these analysis packages, and allow subsequent filtering of results to exclude SNPs on the basis of minor allele frequency and/or number of called genotypes. However, we assume that studies have been otherwise filtered for appropriate quality control metrics to exclude poorly genotyped or imputed SNPs [12].

### Study alignment and error trapping

GWAMA initially checks input data files for errors, such as negative values for odds ratios, and reports any issues to the log file. The study is then excluded from the meta-analysis for that SNP. The reference allele reported in the first study for each SNP is taken as reference, to which all allelic effects are then aligned (Table 1). If studies include estimates of the reference allele frequency, large discrepancies (more than 30%) are reported to the log file for manual checking. If strand information is not provided for studies, GWAMA assumes that alleles are aligned to the forward (+) strand of the NCBI dbSNP database. Strand misspecification is reported to the log file for all non- A/T or G/C SNPs, and are corrected before inclusion in the meta-analysis (Table 1). For A/T and G/C SNPs, strand errors cannot be detected, and all studies are assumed to have provided the correct alignment. However, to overcome potential strand issues for these SNPs, it is recommended that users provide reference allele frequency estimates, so that any large discrepancies between studies can be reported for manual checking.

**Table 1:** Example of alignment of allelic effects and error trapping for a single SNP in a meta-analysis of five studies of a dichotomous phenotype.

Study	Reported strand	Effect allele^1^	Other allele	RAF	Odds ratio (95% confidence interval)	Aligned allelic effect (standard error)	Comment
1	+	A	G	0.12	1.12 (1.07-1.16)	0.11 (0.02)	Allele A taken as reference effect allele.

2	+	G	A	0.85	0.92 (0.87-0.98)	0.08 (0.03)	Effect aligned to allele A.

3	-	T	C	0.12	1.06 (1.02-1.10)	0.06 (0.02)	Effect aligned to allele A on + strand.

4	+	T	C	0.13	1.07 (0.99-1.16)	0.07 (0.04)	Effect aligned to allele A on + strand. Strand error reported to log file.

5	+	A	G	0.87	0.95 (0.90-1.01)	-0.05 (0.03)	Large discrepancy in EAF reported to log file.

^1 ^Effects are aligned to the reference allele in the first study. Errors in the reported strand are recorded in the log file together with warnings regarding potential discrepancies in reported data between studies, for example the aligned reference allele frequency (RAF).

### Fixed-effects meta-analysis

Let *β*_*ij *_denote the strand-aligned effect (log-odds ratio for a dichotomous phenotype) of the reference allele at the *j*th SNP in the *i*th study. The combined allelic effect across all studies at the *j*th SNP is then given by

where *w*_*ij *_= [Var(*β*_*ij*_)]^-1 ^is the inverse of the variance of the estimated allelic effect in the *i*th study, obtained from the standard error (or 95% confidence interval of the odds ratio for a dichotomous phenotype). Note that if the *j*th SNP has not been directly genotyped or imputed as part of the *i*th study, *w*_*ij *_= 0. The variance of the combined allelic effect across studies is given by . Furthermore, the statistic  has an approximate χ^2 ^distribution with one degree of freedom, and this provides the basis of a test of association of the trait with the *j*th SNP over all studies.

### Correcting for population structure

The presence of population structure in a GWA study, not taken account of in the analysis, can lead to over-dispersion of the corresponding association test statistics. One approach to combat this problem is to correct test statistics by the genomic control inflation factor. This factor is given by the median of the test statistics, divided by its expectation under the null hypothesis of no association, which is 0.456 in the context of an allelic-effect based analysis [11]. Users have the option to correct each study for potential population structure, hence the genomic control inflation factor is calculated separately for directly genotyped and imputed SNPs, denoted *λ*_*Di *_and *λ*_D*i_, respectively, for the *i*th study [4,15]. The variance of each SNP in the study is then inflated by the relevant genomic control inflation factor so that , where *K *is replaced by *D *or D*, as appropriate. Furthermore, users have the option of correcting for between-study variation across the meta-analysis so that . In this expression, *λ *is the genomic control inflation factor over all meta-analysed association test statistics, genome-wide.

### Testing for heterogeneity between studies

To test for consistency of allelic effects across studies at the same SNP, GWAMA calculates two summary statistics of heterogeneity [16]. Cochran's statistic  provides a test of heterogeneity of allelic effects at the *j*th SNP, and has an approximate χ^2 ^distribution with *N*_*j*_-1 degrees of freedom under the null hypothesis of consistency where *N*_*j *_denotes the number of studies for which an allelic effect is reported. An alternative statistic, , quantifies the extent of heterogeneity in allelic effects across studies, over and over that expected by chance, and is more robust than *Q*_*j *_to variability in the number of studies included in the meta-analysis [17,18].

### Random effects meta-analysis

In the presence of heterogeneity of allelic effects between studies, it is common to perform random-effects meta-analysis in order to correct the deflation in the variance of the fixed-effects estimate [19]. The random-effects variance component at the *j*th SNP is given by

and is used to inflate the variance of the estimated allelic effect in each study. The combined allelic effect across all studies at the SNP is then given by

where . The variance of the combined allelic effect across studies is given by . As in the fixed-effects meta-analysis, the statistic  has an approximate χ^2 ^distribution with one degree of freedom, and this provides the basis of a test of association of the trait with the *j*th SNP, allowing for heterogeneity of allelic effects between studies.

### Output and analysis summaries

For each SNP, GWAMA will output a variety of summary information and statistics: (i) reference allele to which effects have been aligned and the corresponding non-reference allele; (ii) meta-analysis allelic effect estimate and standard error (or odds ratio and 95% confidence interval for a dichotomous phenotype); (iii) meta-analysis association test statistic, and corresponding *p*-value; (iv) heterogeneity test statistics *Q *(with *p*-value) and *I*^2^; (v) heterogeneity summary, where each study is coded as '+' for increased effect of the reference allele, '-' for decreased effect of the reference allele, '0' for no effect of the reference allele, at a pre-specified significance threshold, and '?' if the study did not report an effect for the SNP. The output from GWAMA can be used with R scripts, supplied with the software, to generate QQ and Manhattan plots to summarise the genome-wide meta-analysis.

## Results

To demonstrate the utility of GWAMA, we present the results of an example meta-analysis of 5 GWA studies of a simulated quantitative trait with directly typed and imputed genotypes at almost 2.4 million SNPs. Association summary statistics for each individual had previously been corrected for population structure, prior to meta-analysis. Statistical tests of association from the fixed-effects meta-analysis at each SNP were corrected for potential between-study variation on output using genomic control. The analysis was completed in just 3.5 minutes using a dedicated processor with 2.4 Gb memory. The data set used in this example is made available with GWAMA to test individual processor capabilities and potential limitations. To evaluate the memory capacity and program running time, we made additional testing with 20, 50, 100 and 200 genome wide datasets (each containing 2.4 million markers). The GWAMA program peaked with memory usage 4.8 GB, 8.2 GB, 14.6 GB, and 26.2 GB accordingly taking 10 min, 24 min, 53 min, and 1 hour 52 min to run.

**Figure 1:**
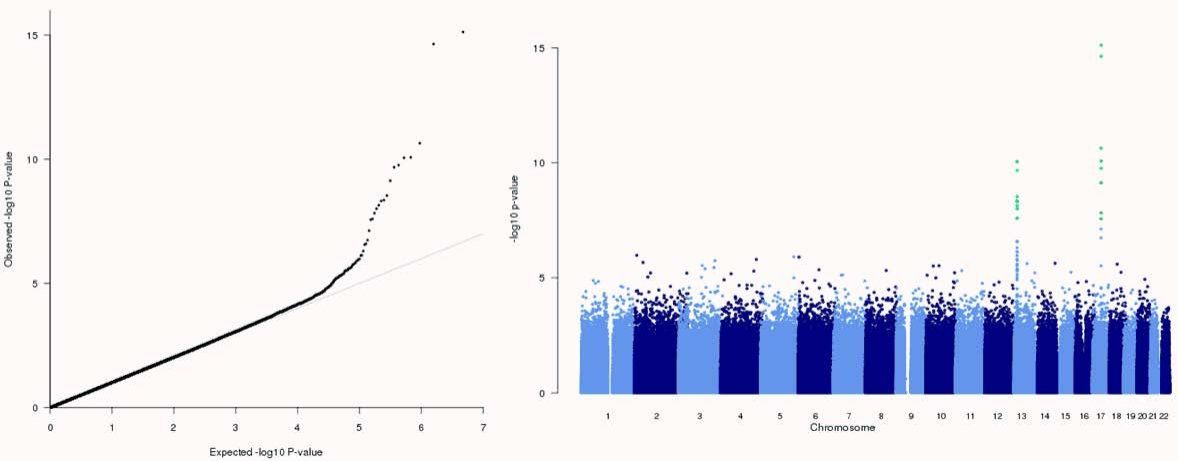
**QQ and Manhattan plots generated from GWAMA output using the summary R scripts released with the software**.

Figure 1 presents QQ and Manhattan plots generated from GWAMA output using the summary R scripts released with the software. The QQ plot indicates that there is no evidence of population structure or between-study variation that has not been accounted in the analysis through genomic control. The Manhattan plot highlights two regions of association, on chromosomes 13 and 17, meeting genome-wide significance (SNPs in green have meta-analysis *p*-value less than 10^-8^).

## Discussion

There are currently several software packages designed for genome-wide meta-analysis of association test statistics including METAL [20], MetABEL [21] and META [22]. Table 2 presents a comparison of the key features of these software packages and GWAMA. The most important advantages of GWAMA over the existing packages are: (i) the distribution of supplementary scripts with the software to allow pre-processing of study summary statistic files generated by widely-used GWA analysis tools and production of graphical summaries to visualise the results of the meta-analysis; (ii) the calculation of two measures of heterogeneity of allelic effects between studies; (iii) the option to perform random-effects meta-analysis is the presence of heterogeneity; and (iv) genomic control correction of the association results of each study, and the meta-analysis overall, to allow for population structure.

**Table 2:** Comparison of software packages for genome-wide meta-analysis of association summary statistics.

***Software package***	METAL	MetABEL	META	GWAMA
Pre-processing of GWA analysis files	No	*ABEL	SNPTEST	SNPTEST, PLINK

Strand flipping for aligning effect directions	Yes	Yes	Yes	Yes

Fixed effect analysis	Yes	Yes	Yes	Yes

Random effect analysis	No	No	Yes	Yes

Heterogeneity statistics (Cochran's *Q *statistic, *I*^*2*^)	*Q*	No	*Q*, *I*^*2*^	*Q*, *I*^*2*^

Automated genomic control for population structure	Yes	Yes	Yes	Yes

Graphical visualisation of meta-analysis results	No	Forest plot	No	Separate scripts for Manhattan and QQ plots

## Conclusions

In the coming months, we expect many more meta-analyses to be undertaken of increasing numbers of GWA studies of a wide range of phenotypes. With the imminent release of data from the 1000 Genomes project [8], we expect imputation to be performed at many millions of SNPs, generating ever larger sets of association summary statistics for analysis. GWAMA is designed to efficiently address the computational challenges of working with such large data-sets by filtering the necessary summary statistics from standard output files from GWA analysis software, as described above. Therefore, we expect that GWAMA will contribute to the identification of novel loci contributing effects to complex human traits in this exciting period of genetic research.

## Availability and requirements

*Project name*: GWAMA

*Project home page*: http://www.well.ox.ac.uk/GWAMA

*Operating system*: UNIX (source code can be compiled with other platforms), Windows XP and newer

*Programming language*: C++, R, PERL

*Other requirements*: C++ compiler, optionally R version 2.9.0 or later with PNG support to generate graphics and PERL to run file formatting scripts

*Licence*: BSD

*Any restrictions to use by non-academics*: none

## Authors' contributions

RM scripted and tested the software. APM provided statistical support and participated in error checking. Both authors wrote and approved the final manuscript.
